# Outcomes and potential impact of a virtual hands-on training program on MRI staging confidence and performance in rectal cancer

**DOI:** 10.1007/s00330-023-10167-4

**Published:** 2023-08-30

**Authors:** Najim El Khababi, Regina G. H. Beets-Tan, Renaud Tissier, Max J. Lahaye, Monique Maas, Luís Curvo-Semedo, Raphaëla C. Dresen, Joost J. M. van Griethuysen, Stephanie Nougaret, Geerard L. Beets, Baukelien van Triest, Stuart A. Taylor, Doenja M. J. Lambregts

**Affiliations:** 1 Department of Radiology, The Netherlands Cancer Institute, P.O. Box 90203, 1106 BE Amsterdam, The Netherlands; 2 GROW School for oncology and reproduction, University of Maastricht, Maastricht, The Netherlands; 3 Biostatistics Unit, The Netherlands Cancer Institute, Amsterdam, The Netherlands; 4 Department of Radiology, Centro Hospitalar E Universitario de Coimbra EPE, Faculty of Medicine, University of Coimbra, Coimbra, Portugal; 5 Department of Radiology, University Hospitals Leuven, Leuven, Belgium; 6 Department of Radiology, UMC Utrecht, Utrecht, The Netherlands; 7 Medical Imaging Department, Montpellier Cancer Institute, Montpellier Cancer Research Institute (U1194), University of Montpellier, Montpellier, France; 8 Department of Surgery, The Netherlands Cancer Institute, Amsterdam, The Netherlands; 9 Department of Radiation Oncology, The Netherlands Cancer Institute, Amsterdam, The Netherlands; 10 Department of Radiology, University College London Hospitals Biomedical Research Centre, London, UK

**Keywords:** Rectal neoplasms, Magnetic resonance imaging, Neoplasm staging

## Abstract

**Objectives:**

To explore the potential impact of a dedicated virtual training course on MRI staging confidence and performance in rectal cancer.

**Methods:**

Forty-two radiologists completed a stepwise virtual training course on rectal cancer MRI staging composed of a pre-course (baseline) test with 7 test cases (5 staging, 2 restaging), a 1-day online workshop, 1 month of individual case readings (*n* = 70 cases with online feedback), a live online feedback session supervised by two expert faculty members, and a post-course test. The ESGAR structured reporting templates for (re)staging were used throughout the course. Results of the pre-course and post-course test were compared in terms of group interobserver agreement (Krippendorf’s alpha), staging confidence (perceived staging difficulty), and diagnostic accuracy (using an expert reference standard).

**Results:**

Though results were largely not statistically significant, the majority of staging variables showed a mild increase in diagnostic accuracy after the course, ranging between + 2% and + 17%. A similar trend was observed for IOA which improved for nearly all variables when comparing the pre- and post-course. There was a significant decrease in the perceived difficulty level (*p* = 0.03), indicating an improved diagnostic confidence after completion of the course.

**Conclusions:**

Though exploratory in nature, our study results suggest that use of a dedicated virtual training course and web platform has potential to enhance staging performance, confidence, and interobserver agreement to assess rectal cancer on MRI virtual training and could thus be a good alternative (or addition) to in-person training.

**Clinical relevance statement:**

Rectal cancer MRI reporting quality is highly dependent on radiologists’ expertise, stressing the need for dedicated training/teaching. This study shows promising results for a virtual web-based training program, which could be a good alternative (or addition) to in-person training.

**Key Points:**

• *Rectal cancer MRI reporting quality is highly dependent on radiologists’ expertise, stressing the need for dedicated training and teaching.*

• *Using a dedicated virtual training course and web-based platform, encouraging first results were achieved to improve staging accuracy, diagnostic confidence, and interobserver agreement.*

• *These exploratory results suggest that virtual training could thus be a good alternative (or addition) to in-person training.*

**Supplementary information:**

The online version contains supplementary material available at 10.1007/s00330-023-10167-4.

## Introduction

MRI plays a crucial part in the diagnostic workup of patients with rectal cancer and is nowadays the standard imaging tool for local staging and to guide treatment planning. Radiologists are an important member of the multidisciplinary team (MDT) where the quality of their diagnostic image interpretation and reporting directly impacts patient management. Several studies have shown that this quality is highly dependent on the radiologist’s experience level and that dedicated training and teaching are essential for radiologists to achieve a diagnostic performance level sufficient to guide MDT decisions [1–4]. In addition to national radiologist training programs, different national and international scientific organizations offer focused rectal imaging courses and workshops, led by expert radiologists. A recent study from Denmark by Bregendahl et al evaluated the impact of various educational elements on the interpretative performance of radiologists in staging rectal cancer on MRI. They showed that it was mainly individual (hands-on) feedback that significantly improved radiologists’ performance, whereas no significant effect was observed after lecture-based teaching workshops or independent case readings only [1]. Though effective, organizing such hands-on face-to-face training courses is a costly, time- and labor-intensive endeavor. The recent COVID-19 pandemic has furthermore had a significant negative impact on the delivery of radiology training and teaching. At the same time, it has given rise to new ways of virtual education. Remote training platforms and virtual teaching alternatives such as webinars and simulated MDTs have rapidly evolved in the past years and will likely continue to form a central component of future education, even now that lockdown restrictions have largely ended [5]. Ideally, the benefits of virtual and hands-on training should be combined into one program, which is why in 2022, we developed a novel virtual hands-on training course focused on MRI of rectal cancer. This training course combines individual case-based training with webinars and online hands-on expert teaching via a newly developed web platform.

In this paper, we have evaluated the outcomes of the first edition of our virtual training course aiming to explore—in a preliminary analysis using a limited number of test cases—the effectiveness of this approach and its potential impact on the diagnostic performance, confidence, and interobserver reproducibility of radiologists in the local staging and restaging of rectal cancer on MRI.

## Methods

This study concerns a retrospective analysis of the outcomes of an international virtual training course focused on MRI of rectal cancer. Course participants provided informed consent to analyze their data as part of this study. The institutional review board of the principal investigating center approved use of the anonymized patient data included in this study, and patient informed consent was waived.

### Course participants and faculty

This virtual training course was designed by the authors and hosted by the European Society of Gastrointestinal and Abdominal Radiology (ESGAR). Sixty-five radiologists from 24 countries participated. The course faculty consisted of seven expert radiologists (R.B-T., D.L., M.L., M.M., R.D., S.N., L.C-S., each with 10–25 + years of experience in clinical rectal MR imaging, rectal imaging research, and education), one dedicated colorectal surgeon (G.B., with > 25 years’ experience in colorectal surgery), and one radiation oncologist specialized in GI oncology (B.v.T., > 20 years’ experience in rectal cancer treatment).

### Virtual training platform (iScore) and teaching materials

For this virtual training course, a dedicated web-based training platform (iScore) was used that was designed by two of the authors (N.E.K.; D.L.). iScore combines the Open Health Imaging Foundation (OHIF) DICOM viewing platform [6] with customizable electronic case report forms (eCRFs) and links to online teaching resources. For this course, the structured reporting templates for rectal cancer MRI (re)staging published by ESGAR were converted into eCRFs and embedded into iScore [7]. For each staging item, a link to a 3–9-min video-lecture was included (12 lectures in total), offering the participants background information and specific staging instructions provided by one of the experts from the faculty. Hyperlinks to relevant background literature [7–12] and educational resources [13, 14] were also included. A visual overview demonstrating the course setup is provided in Supplement 1.

### Case database and online feedback

The case database comprised the MRIs of seven test cases (five primary staging; two restaging after neoadjuvant treatment) and 70 training cases (40 primary staging, 20 restaging, 10 follow-up during organ preservation) that were derived from a previous postgraduate teaching program. Both the test and training cases were selected so that they would offer a clinically representative sample in terms of, for example, T and N stage, mesorectal fascia (MRF) involvement, and extramural vascular invasion (EMVI). For each of the 70 training cases, online feedback forms were constructed. These included the staging templates completed in consensus by two of the expert faculty members (a third reader was consulted to reach consensus in case of any disagreement), accompanied by annotated key images showing the most relevant staging pearls and pitfalls.

### Course setup

**Fig. 1:**
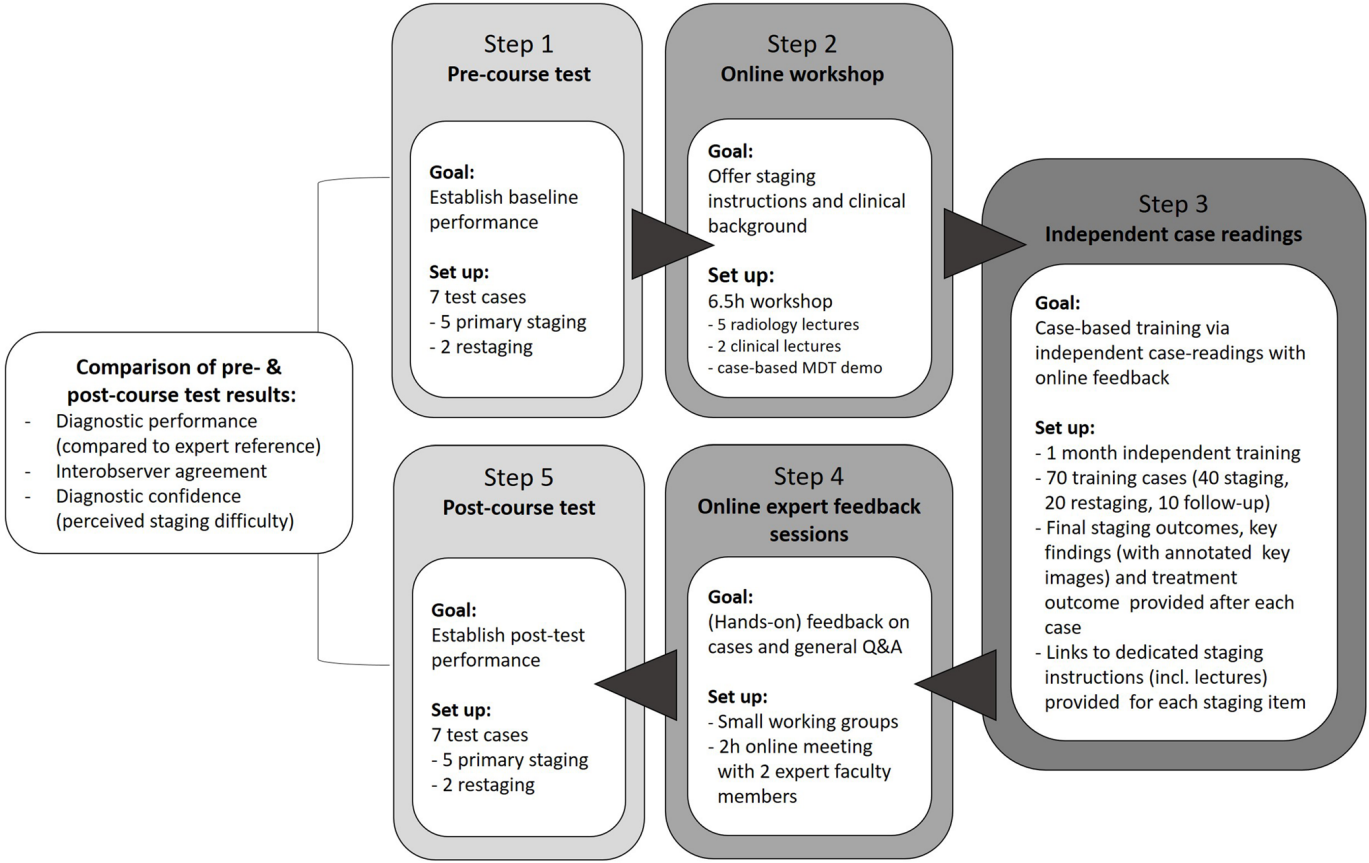
Flowchart demonstrating the five main steps of the virtual training course

The course comprised five main steps (as also detailed in Fig. 1). Prior to the course, participants were asked to provide baseline information regarding their hospital background, level of expertise in reading rectal MRI, and their involvement in colorectal MDT meetings.

1. *Pre-course test*: To establish their baseline performance, participants were asked to complete a pre-course test. This test involved the MRI staging of the seven test cases (5 staging, 2 restaging). For the two restaging cases, the corresponding baseline images were also available for comparison. During this first step, participants did not have access yet to any of the electronic teaching materials, nor did they receive any feedback on their staging outcomes. The participants were asked to indicate for each case whether they found the case easy, moderately easy/difficult, or difficult to stage.
2. *Online workshop*: Participants followed a 6.5-h interactive online workshop including five radiological lectures and two clinical lectures (followed by live discussion and Q&A) and an interactive virtual case-based MDT demonstration.
3. *Independent case readings with online feedback*: After completion of steps 1 and 2, participants got access to the full database of *n* = 70 training cases and all electronic teaching materials. Participants could study these at their own pace over the period of ± 1 month and received feedback after completion of each individual case via the electronic feedback forms described above (see Supplement 1).
4. *Online expert-feedback sessions*: After completing the cases, participants were divided into small groups (± 15 per group) and paired with two expert faculty members per group during a 2-h online discussion session (via Zoom). During these sessions, participants could ask general questions, discuss cases from the case database, and request feedback on their staging errors. Participants were encouraged to send in their questions in advance to allow the faculty to prepare for the sessions and optimize the benefit.
5. *Post-course test:* Within 1 week after completion of the expert-feedback sessions, participants were asked to repeat the staging of the seven test cases (i.e., the same cases previously assessed in the pre-course test). The interval between the pre- and post-course test was > 1 month to avoid recall bias.

Finally, participants were asked to complete a questionnaire regarding user feedback with respect to the course setup, the benefits of virtual versus onsite training, and the use of the virtual teaching platform (iScore).

### Statistical analysis

Statistical analyses were performed using R statistics version 4.1.0 (2021) and IBM SPSS version 27 (2020). Diagnostic accuracy and interobserver agreement (IOA) for each staging item were evaluated for the seven test cases and compared between the pre- and post-course tests. As a standard of reference to calculate accuracy, the seven test cases were scored independently by two of the expert radiologists from the faculty (D.L., R.D.); in case of any disagreement, a third faculty member (M.L.) was consulted to reach final consensus. IOA was calculated using Krippendorff’s alpha. Mixed-effect regression was used to test the course impact on the test-case performance; 2 levels were used for this model (the case number and the course participant) to take into account the multiple measurements per participant and case before and after the course. The responses for the linear mixed model were the absolute difference between the participants’ estimations and the experts. A mixed-effects logistic model was used for binary outcomes, and for continuous outcomes, a mixed-effects linear model where the response was the participant being in agreement with the experts versus the participant being in disagreement with the experts. To evaluate the impact of the participants’ baseline (i.e., pre-course) experience, a model including a covariate representing the level of experience of the reader (< 5 years vs ≥ 5 years’ experience) and an interaction term between the course effect and the experience of the reader is included to test if the effect of the course is different between the different level of experience of the readers. A *p* value of 0.05 was used as the cut-off for statistical significance. Ninety-five percent confidence intervals for Krippendorff’s alpha were computed using bootstrapping.

## Results

### Course participants

Forty-two out of the 65 course participants completed all 5 steps of the course (22 did not complete the post-course test; for 1 participant, no baseline demographics were available). Data of the 42 participants for whom all information was complete were analyzed for the purpose of this study. Table 1 summarizes the participants’ baseline characteristics.

**Table 1:** Baseline characteristics of the 42 course participants who completed the 5 steps of the course

		*n*	%
Total		42	100%
Sex	Male	19	45%
	Female	23	55%
Age median (range)	39 (30–62) years		
Level of expertise	Abdominal radiologist with specific expertise in rectal MRI	4	10%
	Abdominal radiologist	21	50%
	General radiologist	13	31%
	Senior resident	4	10%
	Junior resident	0	0%
Frequency of participating in (colo)rectal MDT meetings	Never	10	24%
	Incidentally	12	29%
	Monthly	11	26%
	Weekly	9	21%
Type of hospital	Comprehensive cancer center	5	12%
	University hospital	19	45%
	General hospital	16	38%
	Other	2	5%
Years after completion of radiology training	Median (range)	7 (0–28)	
	< 5 years	16	38%
	5–10 years	15	36%
	> 10 years	11	26%
Estimated number of rectal cancer cases/MRIs reviewed on a yearly basis	Median (range)	35 (7–300)	
	< 50	24	57%
	50–100	13	31%
	> 100	5	12%
Years of experience in reading rectal MRI	Median (range)	3 (0.5–20)	
	< 1 year	3	7%
	1–5 years	26	62%
	> 5 years	13	31%

### Effect of virtual training on diagnostic performance and interobserver agreement

Table 2 shows the average diagnostic accuracy for the 42 course participants and their interobserver agreement. Though results were largely not statistically significant in our small number of test cases, the majority of variables showed a tendency towards increased diagnostic performance when comparing the pre-course and post-course results. The increase in accuracy ranged between + 2 and + 17%, with the most considerable improvement for assessment of EMVI where mean accuracy improved from 74 to 91%. When looking at the continuous variables (tumor length, tumor height, number of lymph nodes), the difference between the participants and expert reference became smaller for the post-course versus pre-course test, indicating an improved concordance.

**Table 2:** Average diagnostic accuracy (= concordance with expert reference) and IOA for the 42 study participants before and after completion of the virtual training course

Staging variables (derived from ESGAR structured reporting templates)^7^	Average accuracy (min–max)		IOA (Krippendorf’s alpha + 95% CI)		Reference standard (i.e., results of expert reference for the 7 test cases)
	Pre-course	Post-course	Pre-course	Post-course	
Local tumor status					
Morphology—shape (polypoid, annular, semi-annular)	77% (64–98%)	77% (60–95%)	0.64 (0.55–0.73)	0.71 (0.64–0.78)	2 polypoid, 1 annular, 2 semiannular
Morphology—composition (solid, mucinous, mixed)	79% (67–93%)	91% (79–95%)*	0.50 (0.38–0.61)	0.75 (0.65–0.85)**	4 solid, 1 mixed
Tumor length in mm (= average difference in mm compared to expert reference)	9.0 mm (0–52)	7.8 mm (0–47)	0.70 (0.69–0.72)	0.79 (0.78–0.80)**	Range 15–69 mm
Height/distance from anorectal junction in mm (= average difference in mm compared to expert reference)	10.5 mm (0–77)	8.1 mm (0–100)	0.43 (0.41–0.46)	0.57 (0.53–0.60)**	Range 0–49 mm
Response (normalized wall, fibrosis without residual mass, residual mass)^	67% (60–74%)	75% (69–81%)	0.31 (0.17–0.44)	0.52 (0.42–0.62)	1 residual mass, 1 fibrosis without residual mass
T-stage (T0, T1–2, T3ab, T3cd, T4a, T4b)	62% (17–95%)	62% (29–93%)	0.77 (0.73–0.81)	0.76 (0.73–0.79)	1 T0, 2 T1–2, 2 T3ab, 1 T3cd, 1 T4a
Dichotomized T-stage (T0–T3ab, T3cd–T4b)	95% (86–100%)	95% (74–100%)	0.75 (0.61–0.87)	0.79 (0.66–0.91)	5 T0–T3ab, 2 T3cd–T4b
Sphincter invasion (no, yes)	85% (62–100%)	87% (64–100%)	0.44 (0.36–0.52)	0.49 (0.41–0.56)	6 no, 1 yes
Mesorectal fascia and peritoneal involvement					
MRF involvement (no, yes)	86% (62–100%)	90% (57–98%)	0.59 (0.52–0.65)	0.65 (0.59–0.70)	5 no, 2 yes
Relation to peritoneal reflection (above, below, straddling)	79% (43–95%)	85% (66–95%)	0.26 (0.10–0.42)	0.35 (0.19–0.51)	6 below, 1 straddling
Lymph nodes, tumor deposits and EMVI					
N-stage (N0, N +)	80% (36–100%)	82% (40–100%)	0.68 (0.64–0.73)	0.66 (0.61–0.71)	3 N0, 4 N +
Total number of mesorectal nodes (= average difference in no. compared to expert reference)	5.4 (0–18)	4.9 (0–17)	0.58 (0.56–0.61)	0.64 (0.62–0.66)	Range 2–12
Number of suspicious mesorectal nodes (= average difference in no. compared to expert reference	1.1 (0–8)	0.9 (0–7)	0.63 (0.59–0.66)	0.79 (0.77–0.81)**	Range 0–8
Number of suspicious extramesorectal nodes (= average difference in no. compared to expert reference)	0.4 (0–6)	0.3 (0–4)	0.56 (0.62–0.49)	0.69 (0.63–0.74)	Range 0–2
Tumor deposits (no, yes)	84% (26–100%)	91% (48–100%)	0.46 (0.22–0.70)	0.39 (0.01–0.75)	7 no
EMVI (no, yes)	74% (48–100%)	91% (60–100%)	0.49 (0.43–0.55)	0.64 (0.59–0.69)**	5 no, 2 yes

Unless otherwise indicated numbers represent the average agreement for the 42 readers with the expert reference. Results are based on assessment of the seven test cases, including five primary staging cases and two restaging cases after neoadjuvant treatment

^Response was only assessed for the restaging cases

^*^Indicates a significant (*p* < 0.05) difference calculated using a multilogistic regression model (pre-test vs post-test)

^**^Indicates a significant difference based on a 95% CI post-test that does not overlap with the 95% CI pre-test

A similar trend was observed for IOA, which improved for all variables when comparing the pre- and post-course results, except for the assessment of tumor deposits. Improvement in IOA was most evident for morphology (composition), tumor length, distance from the anorectal junction, number of suspicious mesorectal nodes, and EMVI. For these variables, there was no overlap in 95% confidence intervals between the IOA results pre- and post-test, indicating a significant positive effect.

### Impact of participants’ experience level

When comparing the baseline (pre-course) performance of the participants with < 5 versus ≥ 5 years of prior experience in reading rectal MRI, we observed an overall trend towards better concordance with the expert-reference for the more experienced participants (except for T-stage). Results were largely non-significant, except for assessment of tumor length, total number of visible mesorectal nodes, and morphology/composition. For 11/15 staging variables the more experienced readers showed less benefit then the less-expert participants in terms of diagnostic accuracy when comparing the pre- and post-course results, though differences did not reach statistical significance in the vast majority of cases.

### User feedback and effect of virtual training on diagnostic confidence

Table 3 compares the perceived difficulty scores assigned by the 42 participants before and after completion of the course. Overall, there was a significant decrease in the perceived difficulty level (*p* = 0.03), indicating an improved diagnostic confidence. Pre-course, on average, 17% of the cases were perceived as difficult, which decreased to 8% after completion of the course. When asked to rate the usefulness of the current virtual training approach compared to an on-site workshop on a 5-point scale, the average score received by the participants was 3.7 (suggesting a preference for online training).

**Table 3:** Perceived difficulty scores assigned to the seven test cases by the 42 participants

	Pre-test	Post-test
Easy	27%	24%
Moderate	56%	67%
Difficult	17%	8%

Percentages are average scores for the 42 participants multiplied by the 7 test cases (i.e., 294 responses in total). A mixed-effect model was used to test the effect of the course on the perceived difficulty scores. A logistic regression using the pre- and post-course as the main outcome and the difficulty scores as a covariate showed an estimate of − 1.02 (*p* = 0.03)

## Discussion

Using a newly developed web platform for online teaching, a group of 42 international radiologists participated in a dedicated stepwise online training program focused on MRI staging of rectal cancer comprised of online lectures, MDT meeting demonstrations, independent case-based training with online feedback, and online expert feedback sessions Though we fully acknowledge that our study was not powered to perform a detailed analysis or draw any firm conclusions on the merit of our course program on the diagnostic performance of individual staging variables, we did observed some interesting global trends. When comparing the baseline staging results of the participating radiologists to their post-course results, we observed an overall tendency towards an increase in diagnostic staging accuracy ranging from + 2 to + 17%, albeit largely not statistically significant in our small number of test cases. This was accompanied by an improved interobserver agreement and significantly increased staging confidence.

Several previous studies have demonstrated the impact of radiologists’ expertise levels—and hence the importance of dedicated training and teaching—when staging rectal cancer on MRI [1–4, 15]. For example, Rafaelsen et al showed a significant difference in accuracy for T-staging on MRI between an experienced gastrointestinal radiologist (accuracy 88%) and a general radiologist (accuracy 68%) [15]. Our results for individual T-stage assessment were relatively poor (accuracy 62% both pre- and post-training), but results for dichotomized T-stage assessment (T0–3ab vs. T3cd–4) were much better with an accuracy of 95%; these results are in line with previous reports that also show significantly better results for dichotomized T-stage assessment [16, 17].

In a study focused on staging of lateral lymph nodes, Sluckin et al showed that consistency in the anatomical classification and size measurements of these nodes improved for a group of 53 Dutch radiologists after online training sessions led by expert radiologists [2]. In another study by Wang et al, 6 months of targeted training on EMVI was shown to significantly increase the agreement of inexperienced radiologists with an expert reference, as well as the accordance with pathology. The latter study demonstrated a 20% increase in diagnostic performance (using an expert reference), similar to the 17% increase observed in our current study, after a shorter intervention time [3].

When looking at the different individual staging parameters assessed in this study, most evident effects on staging performance and IOA were observed for EMVI, for tumor composition (solid vs mucinous), and for defining the tumor boundaries (i.e., measuring tumor height and length). Interestingly, in contrast with the known inaccuracies of MRI in assessing lymph nodes, results for nodal staging were relatively good with accuracies of 80% pre-course and 82% post-course. When comparing the baseline (pre-course) staging accuracy between participants with < 5 versus ≥ 5 years of prior experience in reading rectal cancer MRI, our results showed a tendency towards a higher baseline accuracy with less effect of the course on staging performance for the more experienced participants, but due to the limited number of cases, most variables were not significant.

The previously mentioned report by Bregendahl et al showed that out of various components of teaching and training, hands-on individual feedback had the most significant impact on staging performance in a group of 18 radiologists and radiology registrars that participated in a dedicated onsite (face-to-face) training program. Their training program—like our virtual course—comprised workshops, independent case readings, and individual feedback sessions [1]. In our current study, we have shown that a similar positive effect as reported by the group of Bregendahl for their face-to-face training program may also be achieved via dedicated online training. In 2022, a working group from the UK Royal College of Radiologists (RCR) assessed and compared the advantages and disadvantages of online versus face-to-face teaching based on experiences prior to and during the COVID-19 lockdown. They concluded that the pandemic had a significant impact on radiology training, with much of the traditional face-to-face training being replaced by remote platforms. While face-to-face training is still essential for procedural training, remote platforms have provided accessibility and have enabled sharing of individual teaching sessions to geographically remote regions. The future of radiology training will likely involve a blend of remote and in-person learning, with virtual teaching remaining a (more) prevalent component.

Setting up effective web-based training and teaching platforms is an important element of success in order to develop high-quality online educational programs. A fast and intuitive DICOM viewing platform is required to offer case-based training. Ideally, such a platform should allow data upload from different sources to share case studies and exchange experiences. In addition, it is essential to give participants a hands-on experience in an online format with dedicated feedback so that they can learn from their mistakes. Individual contact with experienced readers also contributes to the learning experience. With our current course setup, we have tried to bring these elements together. Areas of improvement for the future will be to enable individual case upload and to further expand the level of personal and individualized feedback on a case-by-case basis.

This study has several limitations. First, we cannot rule out a certain selection bias among the study readers considering that participation required a registration fee and will likely have attracted mainly participants with a specific interest (or even preference) for online teaching. The pre- and post-course tests were included in our course setup to allow a first analysis of the outcomes of virtual training, but numbers were deliberately kept small so as not to cause an unnecessary extra burden for the participants. The number of test cases is therefore too small to draw more meaningful and statistically powered conclusions. Moreover, considering the small number of cases, primary staging and restaging results were grouped together while we recognize that these are two distinctly different clinical settings. Also, not all clinical scenarios were represented in the test cases (e.g., no mucinous tumors or cases with tumor deposits were included), which further limits the impact of the study results for these respective staging items. We therefore fully acknowledge that our results must be regarded as exploratory and larger studies should be conducted to further study the effectiveness of online teaching in our current course format. That being said, our results are encouraging and support the ongoing development of online education.

In conclusion, though we fully acknowledge that our results are mainly exploratory, we have shown that a dedicated virtual training course using a web-based platform has potential as an alternative (or addition) to face-to-face training to help radiologists in improving their skills and confidence to stage rectal cancers on MRI.

### Supplementary information

Below is the link to the electronic supplementary material.

Supplementary file1 (PDF 514 kb)

## Abbreviations

eCRF: Electronic case report form
EMVI: Extramural vascular invasion
IOA: Interobserver agreement
MDT: Multidisciplinary team
MRF: Mesorectal fascia

## Declarations

### Guarantor

The scientific guarantor of this publication is Dr. Doenja M. J. Lambregts.

### Statistics and biometry

One of the authors, Mr. Renaud Tissier, has significant statistical expertise.

### Study subjects or cohorts overlap

Some study subjects included in the current cohort have been previously reported on:

• *n* = 5 in a study focused on retrospectively evaluating staging trends in the Netherlands following guidelines updates (Bogveradze et al Evolutions in rectal cancer MRI staging and risk stratification in The Netherlands. Abdom Radiol (NY). 2022;47(1):38–47).

• *n* = 4 in a technical study focused on assessing the reproducibility of quantitative imaging features in multicenter study cohorts (Schurink et al Sources of variation in multicenter rectal MRI data and their effect on radiomics feature reproducibility. Eur Radiol. 2022;32(3):1506–1516).

• *n* = 2 in a study comparing different MRI response evaluation methods in rectal cancer (El Khababi et al Comparison of MRI response evaluation methods in rectal cancer: a multicentre and multireader validation study. Eur Radiol 33(6):4367–4377).

### Methodology

• retrospective

• observational

• multicenter study
