# Supplementary material for: Outcomes and potential impact of a virtual hands-on training program on MRI staging confidence and performance in rectal cancer
Source: Eur Radiol. 2023 Aug 30;34(3):1746–54. doi: 10.1007/s00330-023-10167-4 (PMC10873460; doi:10.1007/s00330-023-10167-4)
Supplement: Supplementary file 1 — Supplementary file1 (PDF 514 kb) [file 330_2023_10167_MOESM1_ESM.pdf]

Supplement 1

The images below (**Figires 1-3)** are screenshots showing the course setup in iScore.

After completion of the pre-course self-assessment test and the virtual kick-off workshop, the participants got access to the full course which included a case database of n=70 cases and links to electronic teaching materials and mini-lectures.

Participants were asked to stage each case using the structured reporting templates from the ESGAR rectal MRI consensus guidelines, which were embedded in iScore as electronic scoring forms. Links to useful background information (scientific and educational papers, websites, etc) were included as blue hyperlinks throughout the scoring forms and links to dedicated mini-lectures on the various staging items were accessible via the logo (**Figure 1**).

Once participants fully completed a case (i.e. answered each staging question), they could save their scorings by clicking "Save" at the bottom of the scoring form. After doing so, a hyperlink entitled "*Click on this link to go to the feedback*" appeared at the bottom of the page (**Figure 2**).

Via this link participants received feedback on their scorings consisting of the final staging outcomes as provided by the expert faculty with accompanying key images demonstrating the main imaging findings, pearls and pitfalls. Participants also received the final treatment and histopathology outcomes (**Figure 3**).

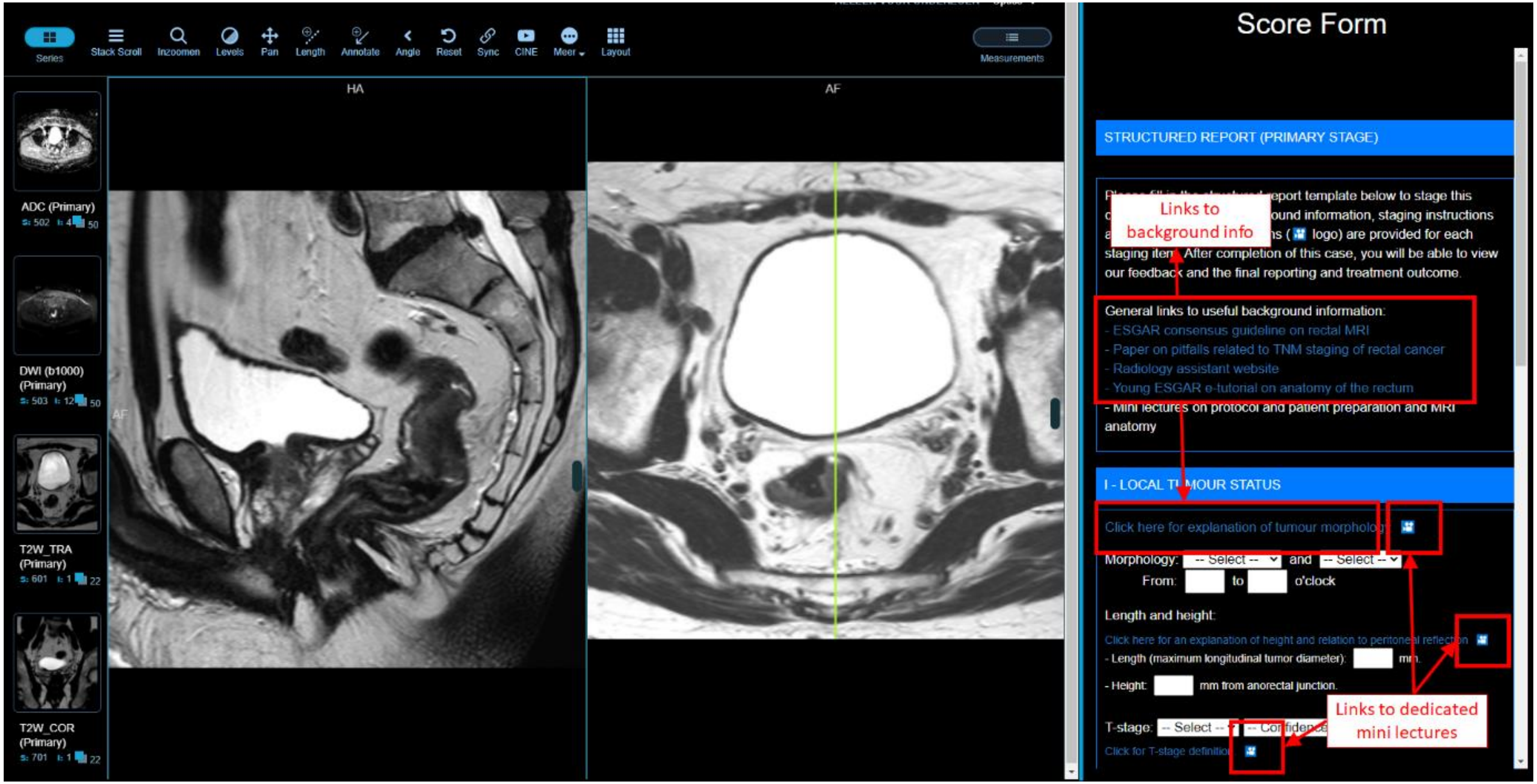
Series
Stack Scroll
Inzoomen
Levels
Pan
Length
Annotate
Angle
Reset
Sync
CINE
Meer
Layout
Measurements
HA
AF
ADC (Primary)
DWI (b1000) (Primary)
T2W_TRA (Primary)
T2W_COR (Primary)
Score Form
STRUCTURED REPORT (PRIMARY STAGE)
Links to background info
logo) are provided for each
After completion of this case, you will be able to view
and the final reporting and treatment outcome.
General links to useful background information:
- ESGAR consensus guideline on rectal MRI
- Paper on pitfalls related to TNM staging of rectal cancer
- Radiology assistant website
- Young ESGAR e-tutorial on anatomy of the rectum
anatomy
Morphology:
-- Select --
and
From:
to
o'clock
Length and height:
- Length (maximum longitudinal tumor diameter):
- Height:
mm from anorectal junction.
T-stage:
Links to dedicated mini lectures

Figure 1

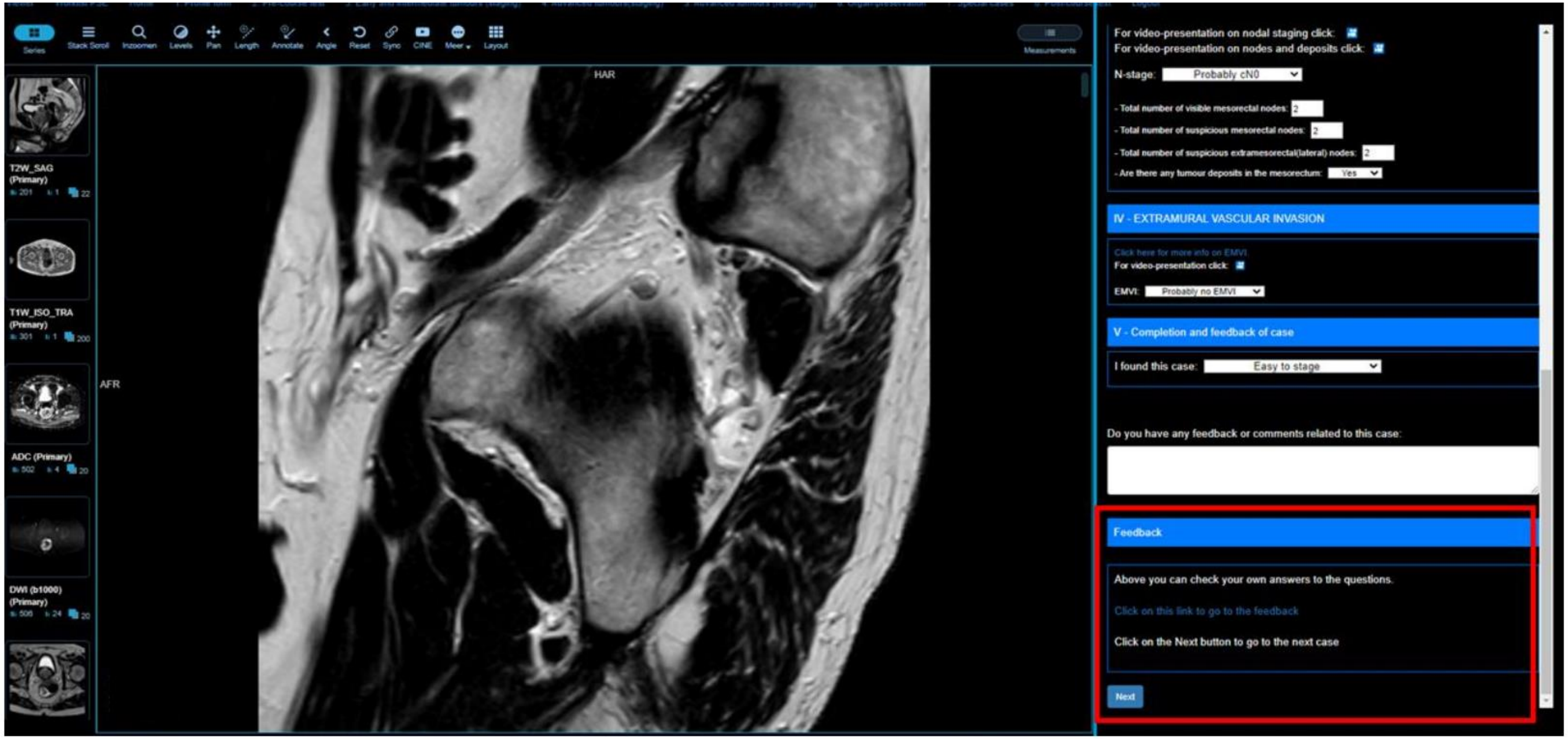

For video-presentation on nodal staging click:
For video-presentation on nodes and deposits click:
N-stage: Probably cN0
- Total number of visible mesorectal nodes: 2
- Total number of suspicious mesorectal nodes: 2
- Total number of suspicious extramesorectal(lateral) nodes: 2
- Are there any tumour deposits in the mesorectum: Yes
IV - EXTRAMURAL VASCULAR INVASION
For video-presentation click:
EMVI: Probably no EMVI
V - Completion and feedback of case
I found this case: Easy to stage
Do you have any feedback or comments related to this case:
Feedback
Above you can check your own answers to the questions.
Click on this link to go to the feedback
Click on the Next button to go to the next case
Next

Figure 2

Feedback Case

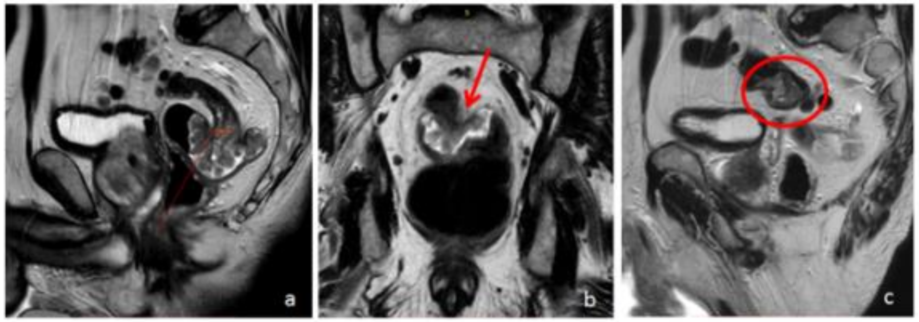

- Polypoid tumour with attachment (stalk) to the rectal wall at the anterior side. The tumour stage is most likely T2 although at the site of the stalk it can be difficult to discern between a T1-2 and limited T3 tumour (see red arrow in image b).
- Please note that there is a second small lesion (cT1-2) more proximal at the level of the rectosigmoid junction (red cirle in c)
- **Final treatment/outcome: TME surgery (pT2N0)**

STRUCTURED REPORT (PRIMARY STAGE)

Local tumour status

- Morphology: ■ Solid - polypoid
  ☐ Solid - (semi-)annular: from .... to .... o'clock
  ☐ Mucinous: from .... to .... o'clock

- Distance from the anorectal junction to the lower pole of the tumour: ±7 cm (see key image a)
- Tumour length: ±5 cm

- T-stage: ■ T1-2
  ▒ T3 → ▒ T3a,b (≤5 mm extramural growth)
  ☐ T3c,d (>5 mm extramural growth)
  ☐ T4, based on growth into: ....

- Sphincter invasion: ■ No
  ☐ Internal sphincter only
  ☐ " + intersphincteric plane
  ☐ " + external sphincter
  } ☐ upper ☐ middle ☐ distal 1/3 of anal canal

Mesorectal fascia (and peritoneal) involvement: Not applicable (T1-2 tumour)

Lymph nodes and tumour deposits

- N-stage: ■ N0 ☐ N+
- Total number of lymph nodes: >8
- Number of suspicious lymph nodes: 0
  ☐ ..... nodes with short axis diameter ≥ 9 mm
  ☐ ..... nodes with short axis diameter 5-8 mm AND <u>at least 2</u> morphologic criteria*
  ☐ ..... nodes with short axis diameter <5 mm AND <u>all 3</u> morphologic criteria*

*N.B. Morphologic suspicious criteria: [1] round shape, [2] irregular border, [3] heterogenous signal

- Are there any tumour deposits within the mesorectum: ■ no,
  ☐ yes, ... (number of deposits)

Extramural vascular invasion

☐ Yes ■ No

Figure 3
